# Cyclosporin a directly binds CCL5: a novel mechanism in SJS/TEN revealed by network pharmacology

**DOI:** 10.3389/fphar.2026.1853184

**Published:** 2026-10-21

**Authors:** Fangqin Peng, Wenjie Hao, Yating Peng, Ougen Liu

**Affiliations:** 1 Department of Dermatology, Nanchang University Second Affiliated Hospital, Nanchang, Jiangxi, China; 2 Department of Health Toxicology, School of Public Health, Shanxi Medical University, Taiyuan, Shanxi, China

**Keywords:** CCL5, cyclosporine A, molecular docking, network pharmacology, SJS/TEN

## Abstract

**Background:**

Stevens-Johnson syndrome/toxic epidermal necrolysis, the most acute and severe of severe cutaneous adverse reactions, has an annual incidence of 0.4–13 cases per million and mortality rates range from <10% in Stevens-Johnson syndrome to >40% in toxic epidermal necrolysis, with an overall mortality of approximately 20%. Cyclosporin A (CsA) is clinically effective in Stevens-Johnson syndrome/toxic epidermal necrolysis (SJS/TEN), but its mechanism beyond calcineurin inhibition remains unclear.

**Objectives:**

To identify novel therapeutic targets of CsA in SJS/TEN using network pharmacology and validate the most promising candidate experimentally.

**Methods:**

Potential CsA targets were predicted using PharmMapper and SuperPred. SJS/TEN-associated genes were retrieved from GeneCards and OMIM. Common targets were analyzed via protein-protein interaction network, Gene Ontology, and Kyoto Encyclopedia of Genes and Genomes enrichment. Molecular docking predicted binding modes. The key target interaction was validated *in vitro* using MicroScale Thermophoresis.

**Results:**

We identified 22 shared targets between CsA and SJS/TEN. Network analysis highlighted five core proteins (MMP9, CASP3, CCL5, CXCR4, CYP3A4). Molecular docking predicted stable binding between CsA and Chemokine (C-C motif) ligand 5 (CCL5). Critically, MST assays demonstrated direct physical interaction between CsA and CCL5 protein with a dissociation constant (Kd) of 2.7 μM.

**Conclusion:**

In conclusion, our *in vitro* experiments confirmed a direct physical binding interaction between CsA and the key chemokine CCL5, providing a novel potential mechanistic hypothesis for the rapid anti-inflammatory effect of CsA in SJS/TEN. However, this hypothesis requires validation through cell-based chemotaxis assays.

## 1 Introduction

Stevens-Johnson syndrome/toxic epidermal necrolysis (SJS/TEN) is a life-threatening mucocutaneous disorder primarily triggered by drugs, characterized by widespread keratinocyte apoptosis and epidermal detachment (Hung et al., 2024). Its mortality varies between <10% in SJS and >40% in TEN (Traikia et al., 2020; Hung et al., 2024). Although the cytotoxic roles of perforin, granzyme B, and TNF-α are well established (Chung et al., 2008), how immune cells rapidly aggregate and sustain effector function within the epidermis remains incompletely understood. Chemokines, particularly the C-X-C motif chemokine ligand 9 (CXCL9), C-X-C motif chemokine ligand 10(CXCL10), are known to recruit cytotoxic T lymphocytes (CTLs) to the skin, but the role of RANTES in SJS/TEN pathogenesis is less explored(Chen et al., 2024).

Cyclosporine (CsA), an 11-amino acid cyclic polypeptide extracted from fungal fermentation products, inhibits calcineurin by blocking the Nuclear Factor of Activated T Cells(NFAT) signalling pathway in T Cells (Lee et al., 2018; Patocka et al., 2021). This reduces the production of cytokines such as interleukin-2, which ultimately suppresses the activation and proliferation of immune cells (Bendickova and Fric, 2020). This drug has been applied clinically in immune-mediated inflammatory dermatological diseases (Rajagopalan et al., 2022). Although it has been repeatedly reported to be effective in treating SJS/TEN (St John J. et al., 2017; Nazerian et al., 2025), its precise mechanism of action in SJS/TEN disease remains unclear.

Chemokine (C-C motif) ligand 5 (CCL5), also known as RANTES, was first identified in 1988 from activated cells and is a classic chemokine (Schall et al., 1988). It mediates the migration of Th1-type cells such as T Cells, monocytes, and macrophages to sites of inflammation, and is secreted by multiple cell types including T Cells, macrophages, epithelial cells, endothelial cells, and tumor cells. A relevant study (Tyner et al., 2005) showed that CCL5 exerts its function by binding to three cell surface receptors: chemokine (C-C motif) receptor 1 (CCR1), chemokine (C-C motif) receptor 3 (CCR3), chemokine (C-C motif) receptor 5 (CCR5). Its biological function is realized by activating downstream signal transduction and transcription factors—specifically the Nuclear Factor κ-Light-Chain Enhancer of Activated B Cells (NF-κβ) and mitogen-activated protein kinase (MAPK) signaling pathways—via these three cell surface receptors.

CCL5, as a chemokine, is extensively involved in processes related to viral infection and the tumor microenvironment, promoting the clearance of viruses or tumor cells by immune cells such as monocytes/macrophages and T lymphocytes. However, some research findings (Gianoukakis et al., 2006; Shi et al., 2022; Li et al., 2025; Xiang et al., 2025) indicate that the overexpressed CCL5 appears to exert a pro-inflammatory effect, exacerbating disease progression or recurrence in autoimmune diseases such as rheumatoid arthritis, systemic lupus erythematosus, multiple sclerosis, and Graves’ disease.

Here, we hypothesized that CsA may exert therapeutic effects in SJS/TEN through direct interaction with extracellular inflammatory mediators, particularly CCL5, independent of its calcineurin-dependent pathway. This study employs network pharmacology and molecular docking techniques to identify potential precise targets of CsA in SJS/TEN and via *in vitro* experiments validate the most promising candidate.

## 2 Materials and methods

### 2.1 Predicting target proteins for cyclosporine A

As shown in Figure 1, the data were downloaded from the following several database. The chemical structure specification (SMILES) for CsA was obtained from the PubChem(https://pubchem.ncbi.nlm.nih.gov) compound database and input into both the PharmMapper (http://lilab-ecust.cn/pharmmapper/index.html) and SuperPred (https://prediction.charite.de) databases to jointly predict potential targets for cyclosporine A. All results were restricted to “human”, the PharmMapper database returned 205 human proteins potentially targeted by CsA. The SuperPred database yielded 141 human proteins potentially targeted by CsA. In total, 334 human proteins were identified as potential targets of CsA.

**FIGURE 1:**
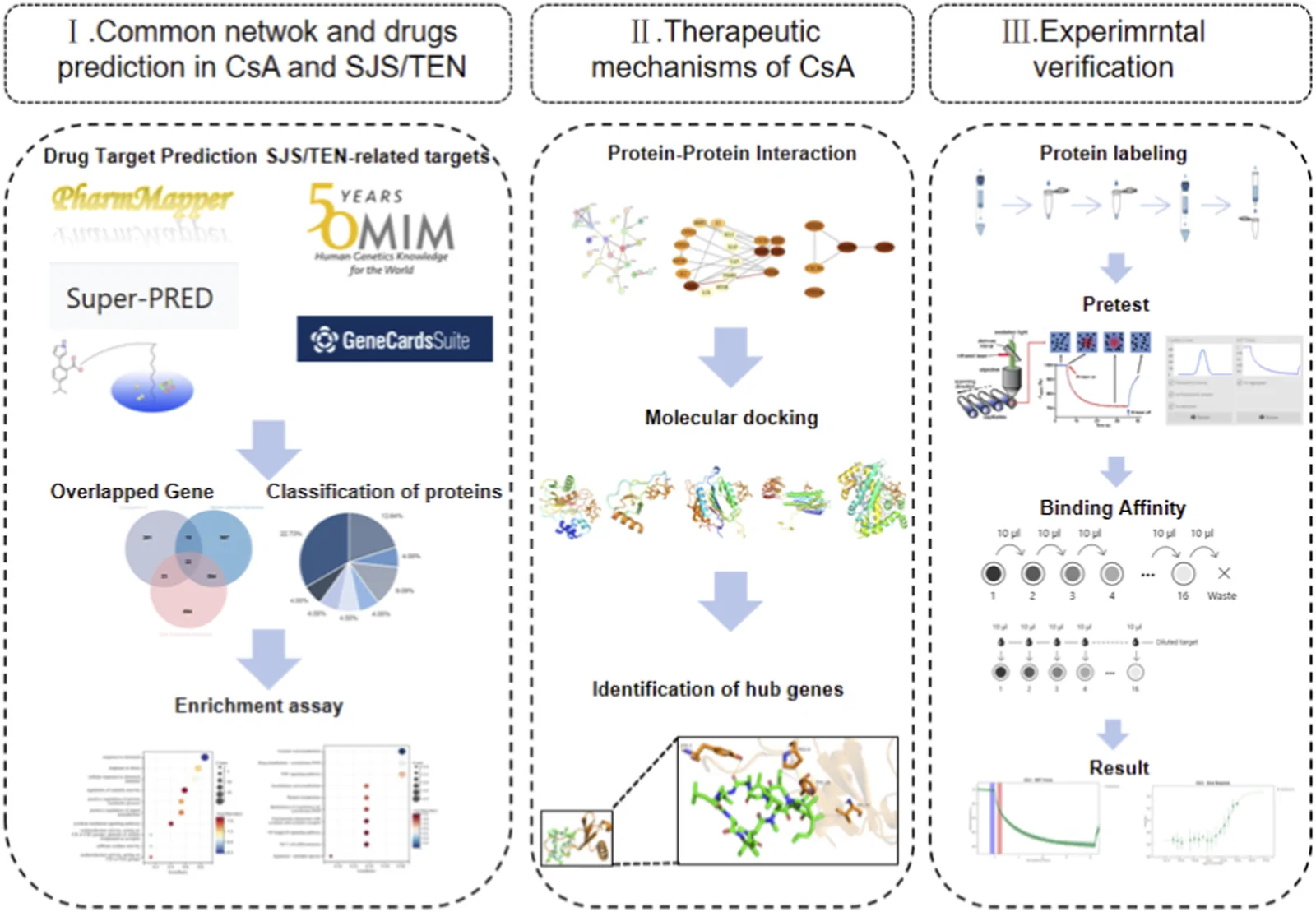
Workflow of the study.

### 2.2 Collecting genes associated with SJS/TEN

SJS/TEN-related targets were retrieved from the GeneCards (https://www.genecards.org/) and OMIM (https://www.omim.org/) databases. Following integration and deduplication of these target sets, 1,211 genes associated with SJS and 1,333 genes associated with TEN were identified, and 22 common targets with cyclosporine A were identified (Table 1; Figures 2a,c-e).

**TABLE 1:** The intersected proteins of Cyclosporin A and SJS/TEN.

Locus	Symbol	Description	Potential role in SJS/TEN
20q13.12	MMP9	Matrix metallopeptidase 9	Protein modifying enzyme
6q21.33	CSNK2B	Casein kinase 2 beta	Protein-binding activity modulator
16q12.2	MMP2	Matrix metallopeptidase 2	Protein modifying enzyme
19q13.41	FPR1	Formyl peptide receptor 1	Transmembrane signal receptor
6q21.32	PSMB9	Proteasome 20S subunit beta 9	Protein modifying enzyme
2q22.1	CXCR4	C-X-C motif chemokine receptor 4	Cell adhesion molecule
1p36.22	mTOR	Mechanistic target of rapamycin kinase	Protein modifying enzyme
7q22.1	CYP3A4	Cytochrome P450 family 3 subfamily a member 4	Metabolite interconversion enzyme
1p34.2	SLC2A1	Solute carrier family 2 member 1	Transporter
4q13.3	ALB	Albumin	Transfer/carrier protein
11p11.2	F2	Coagulation factor II	Thrombin
10q23.33	CYP2C9	Cytochrome P450 family 2 subfamily C member 9	Metabolite interconversion enzyme
Xq25	XIAP	X-linked inhibitor of apoptosis	Protein-binding activity modulator
17q12	CCL5	C-C motif chemokine ligand 5	Intercellular signal molecule
4q27	IL2	Interleukin 2	Transporter
1p35.2	LCK	LCK proto-oncogene, src family tyrosine kinase	Protein modifying enzyme
1p36.13	PLA2G2A	Phospholipase A2 group IIA	Metabolite interconversion enzyme
10q23.33	CYP2C8	Cytochrome P450 family 2 subfamily C member 8	Metabolite interconversion enzyme
1q24.2	SELE	Selectin E	Defense/immunity protein
4q35.1	CASP3	Caspase 3	Protein modifying enzyme
1q13.3	GSTM1	Glutathione S-transferase mu 1	Metabolite interconversion enzyme
6p21.32	TAP1	Transporter 1, ATP binding cassette subfamily B member	Transporter

**FIGURE 2:**
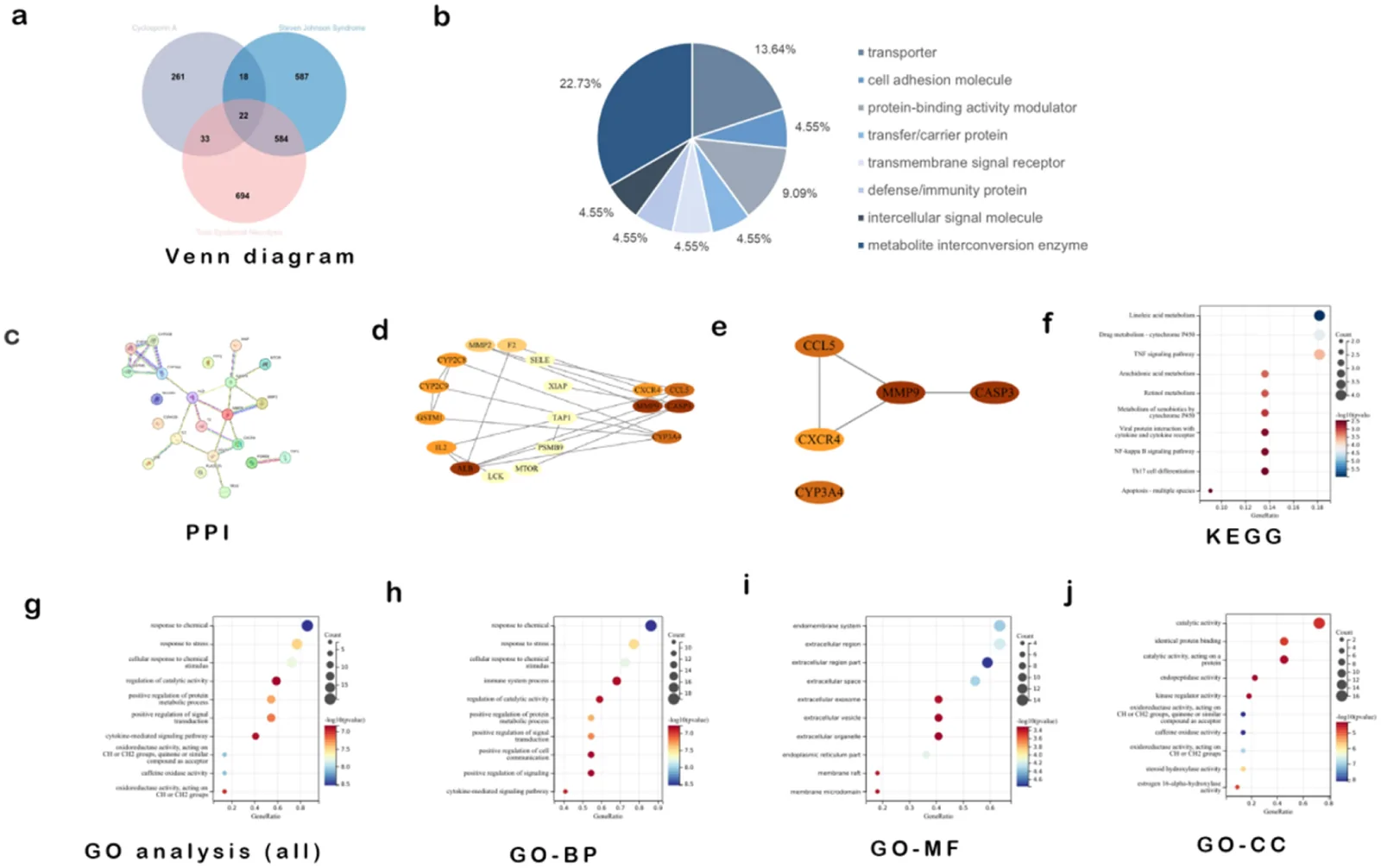
**(a)** Venn diagram of common targets of cyclosporine A (CsA) with Stevens-Johnson Syndrome and Toxic Epidermal Necrolysis. **(b)** The potential targets of CsA. **(c-e)** PPI network of cyclosporine targets. **(f-j)** KEGG and GO enrichment analysis.

### 2.3 Screening of key target proteins and GO and KEGG analyzes

Using www.bioinformatics.com.cn, we identified common genes between CsA and SJS/TEN. The 22 intersecting proteins were imported into the STRING database with a high confidence threshold of 0.7. Eighteen of these proteins were found to interact with each other. Subsequently, the CytoNCA plugin within Cytoscape software (version 3.10.3) was employed to construct a protein-protein interaction (PPI) network. Key targets were screened based on the “Degree”, “Betweenness”, and “Closeness” scores calculated by CytoNCA. Metascape database analysis was performed on the targets for Gene Ontology (GO) and Kyoto Encyclopedia of Genes and Genomes (KEGG) pathway enrichment studies to determine their molecular functions and systemic involvement.

The PPI network of intersection targets between Cyclosporine A,SJS and TEN was constructed from the String (https://string-db.org/). The minimum required interaction score, ranging from 0 to 1, was set to >0.7. The PPI network was further calculated, and hub genes were identified and visual analyzed using Cytoscape software(v 3.10.3). The sangerbox(http://sangerbox.com/home.html) was used to perform GO and KEGG enrichment analyses.

### 2.4 Molecular docking

Molecular docking is one of the key methods in molecular simulation. It constitutes a computational simulation that essentially represents the recognition process between two or more molecules, involving spatial shape complementarity and energy matching between them (Huey et al., 2007; Morris et al., 2009). In the field of molecular modeling, if two molecules can form a stable complex, molecular docking can predict their binding configuration and utilize scoring functions to assess the strength of their interaction based on this configuration (Jumper et al., 2021; Abramson et al., 2024). The software employed for this study is AutoDock Vina, whose algorithm is based on a Monte Carlo search algorithm utilizing local perturbations (Varadi et al., 2024). The ligand-protein docking method employed is semi-rigid docking, where in the ligand is treated as flexible (with rotatable bond angles undergoing free rotation) while the protein is treated as rigid (maintaining fixed conformation). Three-dimensional structural files for the receptor protein are downloaded from the PDB database, followed by structural completion and optimization of the protein structure. Download the 3D structure file for cyclosporine A from the PubChem database and optimize the compound structure. The binding site for this molecular docking reference the compound binding site in the PDB (1U4L) structure. Molecular docking was performed using Autodock Vina. Following processing and optimization of the docking data, analysis was conducted using Discovery Studio Visualizer (version 2024, Dassault Systèmes, San Diego, USA).

### 2.5 Recombinant human CCL5/RANTES protein

Recombinant human CCL5 was purchased from Sino Biological (Cat. #10900-HNAE). According to the manufacturer’s lot-specific certificate of analysis, the protein is ≥95% pure by SDS-PAGE; no additional purification was required before use in the binding experiments.

### 2.6 MicroScale thermophoresis (MST)

The interaction between CCL5 protein and cyclosporine A was determined using MST with the NanoTemper Monolith NT.115 instrument. Recombinant proteins served as targets, fluorescently labeled using the Monolith Protein Labeling Kit RED-NHS (MO-L011, NanoTemper Technologies), with CCL5 acting as the ligand. In each experiment, labeled CCL5 protein (20 nM) was incubated with ligands at varying concentrations (1,000 μM–0.0305 μM) in MST buffer (SD buffer containing 0.05% Tween-20) at room temperature for 15 min. Samples were subsequently loaded into NT.115 standard capillaries. Experiments were conducted at 25 °C with 20% excitation power and 40% MST power (Figures 3d-f). The experimental quality control criterion defines a signal-to-noise ratio greater than 5 as indicative of binding; a ratio exceeding 12 constitutes high-quality data. Protein migration rate diminishes with increasing ligand concentration, yielding an S-shaped fitting curve with distinct upper and lower plateau phases exhibiting pronounced concentration dependence.

**FIGURE 3:**
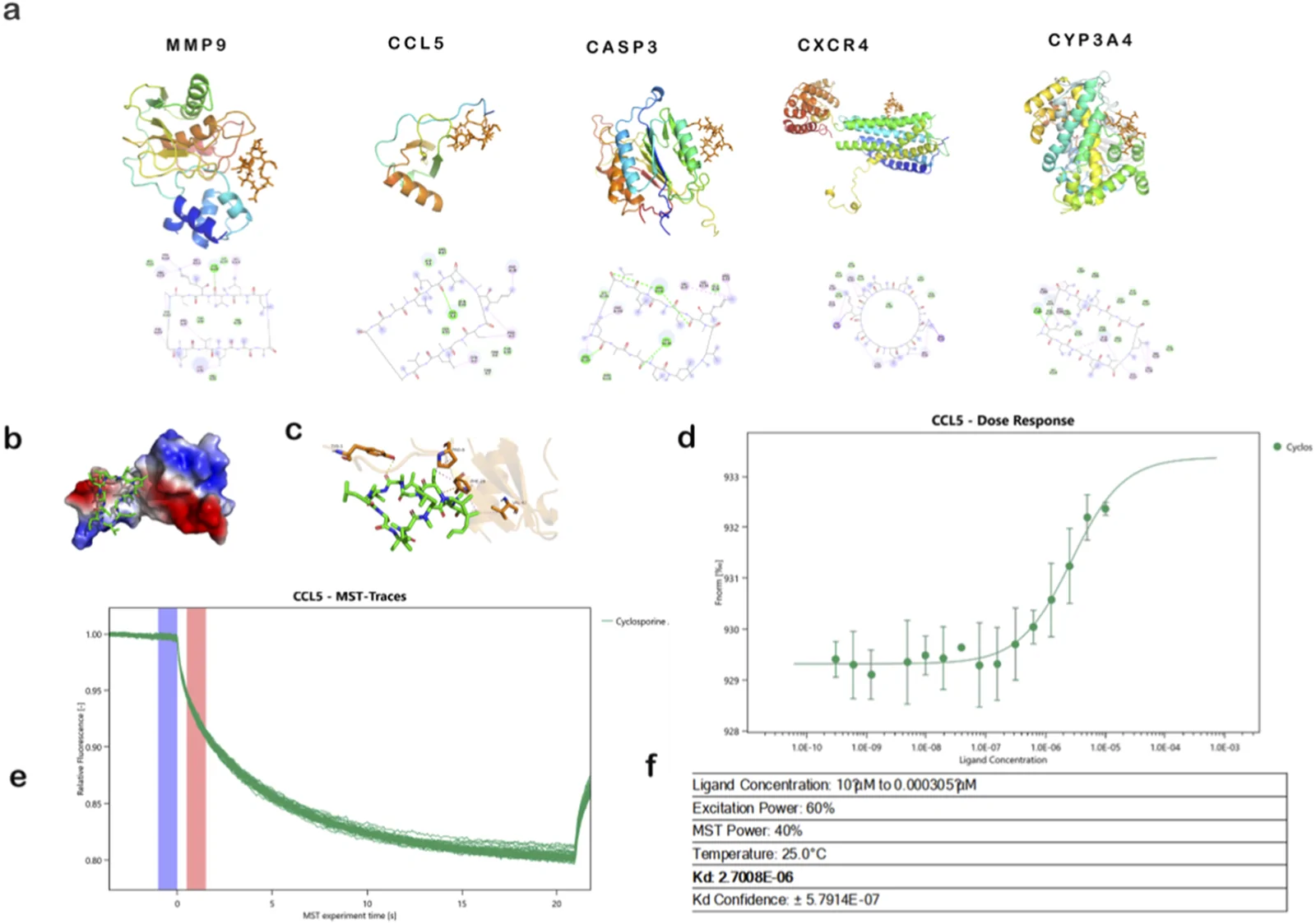
**(a)** Molecular docking revealed the binding of CsA to its targets. **(b)** The presentation of docking results (CCL5) is based on the optimal conformer structure. **(c)** 3D diagram of the interaction between ligand and protein (affinity −5. 8 kcal/mol). **(d-f)** The MicroScale Thermophoresis between CCL5 and CsA.

### 2.7 Statistical analysis

Three independently pipetted measurements were performed for each ligand concentration and pooled into a single dataset. The apparent Kd was obtained by fitting the normalized fluorescence (Fnorm) to a 1:1 binding model using MO Affinity Analysis software (v2.3, NanoTemper Technologies), and the uncertainty is reported as the 95% confidence interval of the fit. The signal-to-noise ratio was 27.3 (>12 indicates high-quality binding data). The apparent Kd of CCL5 for CsA was 2.70 µM (95% CI of the fit: ±0.58 µM). Final data were plotted in Fnorm format using Origin software (v10.2).

## 3 Results

### 3.1 Common target screening and network analysis

From 1,211 genes associated with SJS, 1,333 genes associated with TEN, and 334 human proteins targeted by cyclosporine A, 22 common targets with cyclosporine A were screened and a network diagram constructed (Figure 2a). Key targets were screened based on Degree, Betweenness, and Closeness centrality.

### 3.2 The potential targets of cyclosporine A

Among the 22 proteins (Table 1), protein-modifying enzymes represented the largest category (7 proteins, 33%), followed by metabolic conversion enzymes (4 proteins, 24%), transporters (3 proteins, 14%), and signaling molecules and receptors (4 proteins), with the remainder consisting of signal receptors, adhesion molecules, immune-related proteins, and other functionally diverse subgroups (Table 1; Figure 2b).

### 3.3 GO analysis and KEGG analysis

GO analysis indicates that these proteins are primarily involved in protein metabolism, signal transduction, regulation of catalytic activity, components of the extracellular region, and oxidoreductase activity (Figures 2f-j). KEGG analysis reveals their participation in pathways including linoleic acid metabolism, drug metabolism, tumor necrosis factor signaling, NF-κβ signaling, and apoptosis (Figures 2f-j).

### 3.4 Molecular docking

Molecular docking was performed to identify the possible binding between CsA and five core targets. The results showed that CsA could bind to MMP9, CCL5, CASP3, CXCR4, CYP3A4, with docking scores of −5.4 kcal/mol, −5.8 kcal/mol, −5.3 kcal/mol, −4.7 kcal/mol, and −7.3 kcal/mol, respectively (Figure 3a). During the target identification process, although there was a molecular docking interaction between CYP3A4 and cyclosporine A, this study focused on therapeutic targets rather than metabolic targets. CYP3A4, as an enzyme involved in phase I metabolism, primarily contributes to the elimination of drugs and does not directly mediate the pharmacological effects of cyclosporine A (such as immunosuppression and anti-inflammatory effects). Therefore, it was excluded from subsequent analyses. Following the exclusion of CYP3A4, molecular docking was performed for the remaining four topologically connected targets. The binding energies revealed that CCL5 exhibited the lowest value, indicating the most stable and favorable binding interaction with cyclosporine. Accordingly, CCL5 was prioritized as the primary target for subsequent experimental validation.

### 3.5 Microscale thermophoresis (MST)

To validate this hypothesis, we first employed molecular docking simulations to assess the binding potential between CsA and CCL5. Results demonstrated that cyclosporine stably binds to a structurally accessible hydrophobic pocket on the surface of the CCL5 protein (Figure 3c). This suggests a potential physical interaction between the two.

Furthermore, we employed MST to assess the binding affinity between CsA and recombinant human CCL5 protein under *in vitro* conditions. Experimental results demonstrate a measurable binding interaction between CsA and CCL5, with a dissociation constant (Kd) of 2.7 μM (Figures 3d-f). This indicates that the two substances can undergo direct binding within physiologically relevant concentration ranges.

## 4 Discussion

SJS/TEN is a severe, potentially fatal drug-related cutaneous disorder. Increasing evidence points to an autoimmune mechanism involving Th1-mediated delayed-type hypersensitivity syndrome. CsA, a calcineurin inhibitor, is an effective immunosuppressive agent that selectively blocks cellular proliferation, differentiation, and cytokine production (such as interleukin-6 and tumor necrosis factor-alpha). CsA has been demonstrated to be an effective therapeutic agent for SJS/TEN by suppressing the activity of CD8^+^ T Cells surrounding skin lesions. However, the precise target mechanisms of CsA in treating SJS/TEN remain incompletely elucidated. This study employs network pharmacology methods to investigate potential therapeutic targets for CsA in managing SJS/TEN.

The 22 common targets identified by PPI network screening, along with the five most centrally connected core proteins (Figures 2c-e) —MMP9, CASP3, CCL5, CXCR4, and CYP3A4—constitute a multifunctional network involving inflammatory responses, apoptosis, metabolic regulation, and tissue remodeling. The findings of this study are highly consistent with recent research into the pathogenesis of SJS/TEN. The latest research finding (Olsson-Brown et al., 2023) indicate that MMP9 is closely associated with type IV collagenase and mediates its enhanced activity, participating in the liquefactive degeneration of the basal layer and the process of epidermal necrosis and dissolution in SJS/TEN. CASP3 is the terminal effector that executes keratinocyte apoptosis (Panayotova-Dimitrova et al., 2015).

The upregulation of CCL5 and CXCR4, two members of the chemokine system, is a key driver of the homing and infiltration of SJS/TEN immune cells—particularly cytotoxic CD8^+^ T Cells and monocytes/macrophages—into the skin (Ogiji et al., 2024). CYP3A4, a member of the cytochrome P450 superfamily, is a key phase I metabolic enzyme predominantly expressed in the liver and small intestine, where it catalyzes the oxidative metabolism and elimination of a broad spectrum of endogenous and exogenous compounds (Zanger and Schwab, 2013; Wang et al., 2025). Notably, although CYP3A4 exhibited the highest docking score (−7.3 kcal/mol) in our network analysis, it was ultimately excluded from the core target set. In the network pharmacology–based target analysis conducted prior to molecular docking, CYP3A4 was identified as one of five core targets; however, unlike the other four targets, it constituted an isolated node with no topological connections to the remaining core genes. This lack of interaction was the primary rationale for its exclusion: given that CYP3A4 acts independently within the network, its modulation alone would be unlikely to exert coordinated effects on the other four genes, and it therefore did not meet the criteria for a *bona fide* core target. Functional considerations lend further support to this decision. CYP3A4 primarily mediates xenobiotic metabolism and drug elimination *in vivo*, rather than participating in the pathological pathways underlying the disease, rendering it an unlikely direct therapeutic target of cyclosporine.

We acknowledge that the free plasma concentration of CsA is a key consideration for physiologycal relevance of our findings. Following oral administration, total plasma CsA is approximately 100∼400 ng/mL, of which >90% is protein-bound, leaving free concentrations in the nM range. However, plasma levels likely underestimate drug exposure at inflammatory sites: owing to its high lipophilicity and large volume of distribution, CsA preferentially accumulates in the skin, and epidermal concentrations in orally treated psoriasis patients have been reported to be ∼10-fold higher than concurrent trough blood levels (Fisher et al., 1988). Tissue accumulation may therefore elevate the local free CsA concentration at inflammatory sites to the micromolar range, providing a physiologically plausible concentration window for the CsA-CCL5 interaction measured here (Kd = 2.7 μM).

Based on this, we propose the following hypothesis: cyclosporine may directly bind to the CCL5 protein, thereby inhibiting CCL5-mediated chemotaxis and tissue infiltration of immune cells, ultimately alleviating drug rash-associated inflammatory responses. However, this hypothesis requires validation through cell-based chemotaxis assays.

In a fundamental study of drug-induced liver injury induced by acetaminophen, it was discovered that serum and hepatic CCL5 levels were both elevated (Li et al., 2020). CCL5 can activate the MAPK and NF-κβ pathways by binding to CCR1 and CCR5 ligands, suggesting that CCL5 blockade represents a promising therapeutic strategy for drug-induced liver injury. Interestingly, existing research has identified increased CCL5 expression in the serum and blister fluid of SJS/TEN patients, though further investigation is warranted (Chen et al., 2024).

Although this study presents innovative insights, certain limitations remain, pointing towards several key directions for future research. Bioinformatics predictions and combined experiments merely suggest possibilities, yet further validation of target function is still lacking. Subsequent work should validate these predicted targets as functional targets for CsA’s *in vivo* efficacy through experiments in cellular models (e.g., verifying CsA’s effect on keratinocyte death induced by CASP3 activation) and SJS/TEN animal models. This should be achieved using approaches such as gene knockdown/overexpression and specific inhibitor controls. This study confirmed CsA’s *in vitro* binding to CCL5 via MST assays; however, whether this binding causes CCL5 inactivation, enhances its clearance, or affects its dimerization/oligomerization remains unclear. Cell chemotaxis assays are required to directly observe whether CsA pre-incubation blocks CCL5’s ability to chemotaxis T Cells or monocytes. Clinical relevance requires confirmation: Future studies should collect serial serum, blister fluid, and skin biopsy samples from SJS/TEN patients receiving CsA therapy. Dynamic monitoring of key molecular levels—including CCL5, TNF-α, and sFasL—should be conducted and correlated with clinical responses (e.g., time to cessation of skin desquamation, improvement in SCORTEN scores (Fouchard et al., 2000; Noe et al., 2019)) to validate the actual contribution of these mechanisms in patients.

## 5 Conclusion

In summary, this study employed network pharmacology methods to map the potential target network of cyclosporine A within the SJS/TEN context, revealing its multi-targeted, multi-pathway therapeutic potential that transcends the traditional immunosuppression framework by directly intervening in apoptotic pathways, chemokine activity, and metabolic processes. Crucially, our *in vitro* experiments have demonstrated CsA’s direct physical binding with the key chemokine CCL5, offering a novel potential mechanistic hypothesis to explain CsA’s rapid anti-inflammatory effects in SJS/TEN. These findings not only deepen our understanding of the mechanism of action of this classic immunosuppressive agent, but also provide crucial theoretical underpinnings and experimental clues for developing more targeted therapeutic strategies for SJS/TEN based on novel targets such as CCL5.

## Data Availability

The raw data supporting the conclusions of this article will be made available by the authors, without undue reservation.
